# Reduced aggregation of the leghorn male hepatoma cell line in suspension by supplementing dextran sulfate in the media

**DOI:** 10.5713/ab.24.0372

**Published:** 2024-08-27

**Authors:** Jung Soo Lim, Ji Soo Kim, Yugyeong Cheon, Tae Sub Park, Jong Kwang Hong

**Affiliations:** 1 Division of Biological Science and Technology, Yonsei University, Wonju 26493, Korea; 2 Graduate School of International Agricultural Technology and Institute of Green-Bio Science and Technology, Seoul National University, Pyeongchang 25354, Korea

**Keywords:** Cell Adhesion Molecules, Cell Aggregation, Dextran Sulfate, Leghorn Male Hepatoma (LMH), Suspension

## Abstract

**Objective:**

The study aimed to improve the efficiency of leghorn male hepatoma (LMH) cells for animal virus vaccine production by transitioning from adherent to suspension culture and evaluating the effects of dextran sulfate (DS) on preventing cell aggregation. The goal was to enhance cell growth, viability, and glucose metabolism and to develop efficient suspension-adapted LMH cells for large-scale vaccine production.

**Methods:**

LMH cells previously cultured in an adherent state were transferred to 125 mL Erlenmeyer flasks to conduct suspension culture. Cell culture performance, including cell density, viability, and glucose metabolism, during the cultures was measured, along with an assessment of cell aggregation. Additionally, mRNA expression levels of genes associated with cell adhesion and apoptosis were monitored.

**Results:**

DS supplementation in suspension culture enhanced cell viability and growth, with higher cell densities and viabilities compared to control media. Additionally, DS supplementation reduced glucose consumption and waste production, indicating improved metabolic efficiency. DS also delayed cell aggregation, possibly by downregulating integrin expression and promoting anti-apoptotic gene expression. However, even after 2 months, cell aggregation persisted in both control and DS-supplemented cultures, suggesting further optimization is needed for LMH cell adaptation to suspension culture.

**Conclusion:**

DS supplementation in LMH cell suspension cultures led to notable improvements in cell growth, viability, and glucose metabolism, while also decreasing the cell aggregation.

## INTRODUCTION

Animal cell lines are crucial as cell factories for biopharmaceutical production, such as therapeutic proteins and vaccines [1]. While Chinese hamster ovary (CHO) and human embryonic kidney 293 (HEK293) cells are primary hosts for producing human therapeutic proteins and viral-based products, many other immortalized animal cell lines such as Madin-Darby canine kidney (MDCK), Vero, Medical Research Council cell strain 5 (MRC-5), chicken embryo lung (CEL), and leghorn male hepatoma (LMH) are also utilized for viral-based vaccine production [2–6]. Achieving large-scale commercial production with these animal-derived host cells requires high-density and easily up-scaled cell cultivation. Traditionally, large-scale cultivation of animal cells, which generally proliferate in an attached state, utilized roller bottles, multi-layered cell factory flasks, or the scale-out approach employing multiple flasks to increase the attachment surface area [7,8]. However, these methods have drawbacks, including time-consuming steps like trypsin treatment for cell detachment, which also increases the risk of contamination, thereby limiting the production efficiency.

To overcome these challenges, there is a growing focus on adapting animal cells from attachment to suspension cultivation, which simplifies sub-culture and reduces the constraints on scale-up, as it does not require trypsin treatment or medium exchange. This approach is particularly critical for vaccine development in the livestock and poultry industries, where industrial application has been constrained by the need for various animal-derived host cells and the lack of basic research supporting large-scale cell cultivation. In the poultry industry, vaccines are essential for ensuring food security and controlling zoonotic diseases such as avian influenza. Therefore, optimizing the suspension culture of LMH host cells, which are representative poultry cells, emerges as a pressing technology in poultry vaccine manufacturing research. [9–11].

Many studies have successfully implemented suspension culture of animal cells for human biopharmaceutical production, i.e., suspension cultures of CHO cells for monoclonal antibodies and HEK293 cells for gene therapy [12,13]. In the production of animal therapeutics, however, conventional adherent culture methods are primarily employed. In particular, cells derived from chickens, like DF-1 and LMH, which are crucial for avian virus vaccine development, predominantly undergo adherent culture [14–16]. Considering that avian viruses pose significant economic threats to the poultry industry and potential public health risks through zoonotic transmission [17], there is an urgent need for swift and efficient vaccine mass production. To address this challenge, further exploration of suspension culture is imperative.

Adapting cells to suspension culture is a time-consuming and difficult process that does not guarantee successful adaptation. Moreover, when animal cells grown in an attached state are transferred into the suspension culture, large cell clumps can quickly form due to the molecules involved in cell surface adhesion [18–21]. Since large cell aggregates create imbalances between internal and external cells in terms of nutrient supply and gas exchange, heterogeneous metabolic activities are induced in the bioreactor, decreasing cell viability and productivity and batch-to-batch consistency [22,23]. Therefore, suspension culture without cell aggregates is crucial for improving bioprocess performance. Several methods have been employed to resolve these cell aggregation problems in suspension cultures, such as long-time subcultures in suspension, clonal isolation of fully adapted clones, and media development for reduced cell aggregation [24]. For example, the addition of certain enzymes, polymers, surfactants, and other substances to the medium can prevent cell aggregation [25–28]. In addition, dextran sulfate (DS), a complex polysaccharide, decreases cell-cell adhesion and aggregation in suspension culture [11,29–33]. This effect is attributed to the negative charges on sulfate groups, inducing electrostatic repulsion between these negative charges on the cell surface [34]. Since DS is usually added to the suspension culture media for animal-derived cell lines such as CHO and HEK cells due to its anti-clumping effect, it would be meaningful to explore the inhibitory effects of DS on cell aggregation of avian-derived LMH cells in a suspension culture.

In this study, we investigated the impact of DS in the medium on suspended cell cultures of LMH cells. We observed changes in cellular metabolism, cell aggregation, and expression of cell-cell adhesion- and apoptosis-related proteins to investigate the effects of DS in the medium.

## MATERIALS AND METHODS

### Cell cultures

The adherent LMH cell line was cultured in T-flasks pre-coated with 0.1% gelatin (Sigma-Aldrich, Saint Louis, MO, USA) with Iscove’s modified Dulbecco’s medium (IMDM) supplemented with 4 mM glutamine and 10% (v/v) fetal bovine serum (FBS) (Gibco, Waltham, MA, USA). Flasks were incubated in an ANICELL CO_2_ incubator (N-Biotek, Bucheon, Korea) at 5% CO_2_, 95% humidity, and 37°C. To conduct subculturing or sampling, supernatants were collected, cells in the T-flask were trypsinized, and viable cell density (VCD) and viability were measured.

To conduct suspension culture, LMH cells grown in the attached state were inoculated into 125 mL Erlenmeyer flasks (Corning, Corning, NY, USA) with a working volume of 30 mL and a seeding density of 0.3×10^6^ cells/mL. Suspension flasks were incubated in the same CO_2_ incubator but with a 110 rpm agitation speed. Medium for the control condition was IMDM supplemented with 4 mM glutamine and 10% (v/v) FBS (Gibco, USA). For the DS-supplemented condition, DS of 100 mg/L (Catalog number: 31404; Sigma-Aldrich, USA) was added to the control medium. During suspension cultivation, sampling was performed every 2 days to analyze VCD, viability, and concentrations of metabolites.

In addition, adherent LMH cells were inoculated at a density of 1.0×10^6^ cells/1.5 mL/well in 24-well plates (Corning, USA). These 24-well plates were incubated in a Kuhner shaking incubator (Kuhern Shaker Inc, San Carlos, CA, USA) set to 37°C, humified 5% CO_2_, and 200 rpm.

For long-term subcultures, LMH cells in an Erlenmeyer flask were subcultured for 18 passages at intervals of 3 to 4 days. During each subculture, VCD, viability, and metabolites were measured, and then cells were re-inoculated into a clean Erlenmeyer flask at a concentration of 0.3×10^6^ cells/mL for the next passage.

### Culture analysis

Viable cell concentration and viability of the cultures were analyzed using a Vi-Cell XR cell counter (Beckman-Coulter, Brea, CA, USA). Residual concentrations of glucose, lactate, and ammonia in culture supernatants were measured using an RX Misano (Randox, Antrim, UK). For gene expression level analysis, cells were harvested, washed twice with phosphate-buffered saline, and centrifuged at 10,000 rpm. The supernatant was removed, and cell pellets were stored in −80°C in a deep-freezer for later analysis. Microscopy images of cells on a 24-well plate were captured using an Eclipse TE2000-U microscope (Nikon, Tokyo, Japan) every 24 hours.

### Real-time reverse-transcriptase polymerase chain reaction

The mRNA extraction followed the manufacturer’s instructions for RNeasy Kits (Qiagen, Venlo, Netherlands) using cultured cell pellets. Glyceraldehyde-3-phosphate dehydrogenase (GAPDH) and β-actin served as reference genes. Expression levels of BH3 Interacting Domain Death Agonist (BID), Caspase 9, and BCL-xL were analyzed for cellular apoptosis, while E-cadherin, integrin α2, integrin αV, integrin β1, and integrin α6 were analyzed for cell adhesion. Real-time reverse-transcriptase polymerase chain reaction was performed using the StepOnePlus System (Applied Biosystems, Waltham, CA, USA) with SYBR green. The comparative Ct (ddCt) method was used to calculate the expression levels of genes. Primers used in this study are shown in Table 1.

### Statistical analysis

Results of all experiments are expressed as mean±standard error of the mean. The statistical significance of the compared data sets was analyzed by paired, parametric test. Paired t-test or multiple-paired t-tests were performed using Graphpad Prism 9 software (Graphpad Software, Boston, CA, USA). Results of p-values which were set at p<0.05, considered to be significant in the data sets (* p<0.05, ** p<0.01, *** p<0.005).

## RESULTS

### Culture profiles of attached leghorn male hepatoma cells in T-flask cultivation

**Figure 1:**
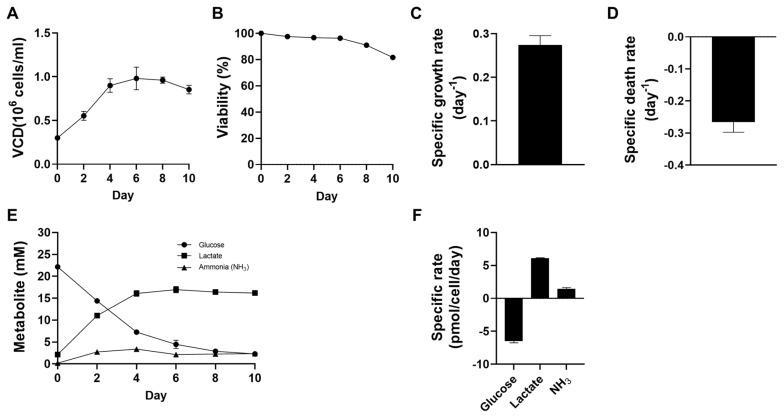
The culture performance of LMH cells in adherent culture. LMH cells were inoculated into 0.1% gelatin pre-coated T25 flasks with a seeding density of 0.3×10^6^ cells/mL. Sampling was conducted every 2 days. (A) VCD; (B) viability; (C) specific growth rate during days 0 to 4, (D) specific death rate during days 0 to 4; (E) concentrations of glucose (circle), lactate (square), and ammonia (triangle) of the culture supernatants; (F) specific rate of metabolite during days 0 to 4. Error bars indicate standard deviation calculated from three independent experiments. LMH, leghorn male hepatoma; VCD, viable cell density.

Before investigating the impact of DS supplementation on the suspension culture performance of LMH cells, we first examined the inherent cell culture performance of LMH cells in an adherent state (Figure 1). LMH cells were inoculated into gelatin-coated T-25 flasks at a concentration of 0.30×10^6^ cells/mL to perform batch culture, where cell growth, viability, nutrient, and waste concentrations were measured every two days. Most viable cells were on the surface of the flask, although 10% of them were present in the culture medium (data not shown). Therefore, cell density and viability were considered for both cells attached to the flask and those present in the culture medium. During batch culture, a maximum cell density of 0.98±0.11 ×10^6^ cells/mL was observed on day 6. The specific growth rate during days 0 to 4 was 0.27±0.02 day^−1^, lower than that of CHO cells [35], which are commonly used for protein production. After reaching the peak VCD on day 6, a decrease in viability was observed for days 6 to 10, reaching 81.60%±0.83% on day 10. The glucose in the initial medium was continuously consumed at a rate of glucose consumption of −6.46±0.30 pmol/cell/d (days 0 to 4). After peak VCD on day 6, glucose concentration dropped below 5 mM, establishing a correlation between cell death and low glucose concentration. Lactate, a major waste product of cell culture, was produced at a production rate of 6.05±0.13 pmol/cell/d until day 4. After a maximum concentration of 17.0 mM on the 6th day, lactate concentration was maintained. Another major waste product, ammonia [27,36], was maintained at levels below 3.9 mM throughout the culture period.[Table t2-ab-24-0372]

### Effect of dextran sulfate on the culture performance of leghorn male hepatoma cells in suspension

**Figure 2:**
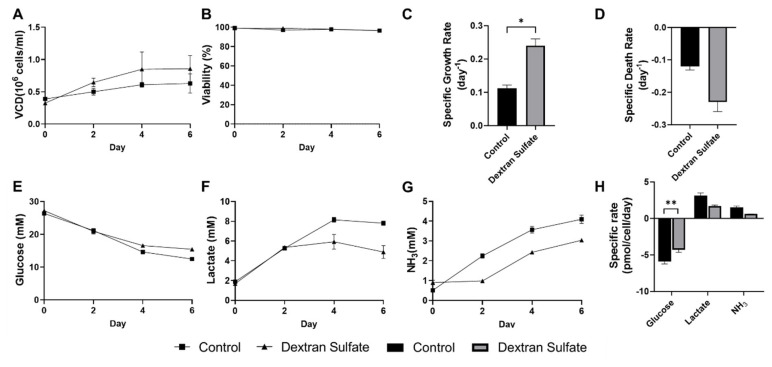
The culture performance of LMH cells in suspension culture. Exponentially growing LMH cells were inoculated in a 125 mL Erlenmeyer flask with the VCD of 0.3×10^6^ cells/mL, and sampling was conducted every two days. Working volume and agitation speed were 30 mL and 110 rpm respectively. (A) Viable cell density; (B) viability; (C) specific growth rate (day 0 to day 4); (D) specific death rate (day 0 to day 4); (E) residual concentrations of glucose; (F) lactate; and (G) ammonia (triangle) of the culture supernatants; and (H) specific production rates of metabolites. Error bars indicate standard deviation calculated from three independent experiments. LMH, leghorn male hepatoma; VCD, viable cell density. Paired t-test was performed to analyze statistical significance (* p<0.05, ** p<0.01).

**Figure 3:**
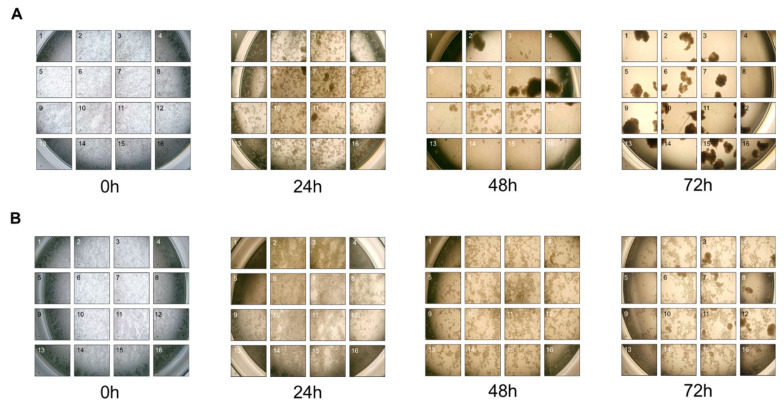
Time-lapse photo images of LMH cell aggregation in suspended 24-well plate cultures. LMH adherent cells were inoculated into a 24-well plate at a density of 1×10^6^ cells/1.5 mL/well, followed by suspension cultures with an agitation speed of 200 rpm. The entire area of a single well was captured with 16 photos at 24-hour intervals for 3 days. The formation of cell clumps was delayed in the DS-supplemented medium compared to the control medium, and both the size and number of aggregates decreased. (A) Images of the LMH cells in the control condition; and (B) images of the LMH cells in the DS-supplemented condition. LMH, leghorn male hepatoma; DS, dextran sulfate.

We then investigated the impact of DS supplementation on cell growth and metabolism of the LMH cells in suspension culture derived from an attachment culture. Exponentially-growing LMH cells from T-flasks were inoculated into Erlenmeyer flasks, and suspension batch cultures were conducted in a shaking incubator at 110 rpm (Figure 2). Two types of media were used in this batch culture: i) control medium used for adherent culture; and ii) medium supplemented with DS. Cell density; viability; and glucose, lactic acid, and ammonia concentrations were measured at 2-day intervals after inoculation. In both control and DS-supplemented media, significant cell aggregation was observed over time, as shown in Figure 3. To accurately determine the VCD, the cell culture broth prepared for sampling underwent 1 hour of trypsinization to dissociate the aggregated cells. However, this method did not fully dissociate cell clumps into single cells, complicating the measurement of cell density, particularly after day 4. Despite this, incorporating DS into the medium enhanced the viability of LMH cells in comparison to the control medium.

DS supplementation exhibited a positive impact on the performance of LMH cell cultures (Figure 2). When DS was added to the medium, VCD reached 0.66±0.07 ×10^6^ cells/mL with a viability of 99.0%±0.0% on day 2, surpassing the 0.50±0.05 ×10^6^ cells/mL and 97.1%±0.0% respective values of the control. Similarly, by day 4, the VCD and viability in the DS-supplemented condition (0.85×10^6^ cells/mL and 98.1%±0.0%) were higher compared to the control (0.6.1± 0.0 ×10^6^ cells/mL and 97.9%±0.0%). Throughout the cultures, spent media were collected and analyzed for glucose, lactic acid, and ammonia concentrations. Notably, DS supplementation led to reduced glucose consumption and lower production of the waste products lactate and ammonia. This result suggests that adding DS to the media may enhance cell metabolism efficiency, resulting in improved cell growth and viability while utilizing less glucose and generating smaller amounts of waste products.

### Effect of dextran sulfate on the aggregation of leghorn male hepatoma cells in suspension

In both suspension culture conditions above, even though single cells were initially inoculated, a noticeable formation of cell clumps was observed as the cultures progressed, suggesting rapid increase of cell-to-cell interaction. Interestingly, the degree of cell aggregation was significantly reduced in the DS-supplemented medium (Data not shown), leading to enhanced culture performance in terms of cell growth and viability (Figure 2). To evaluate the process of cell aggregation in suspension cultures, cells were inoculated into a 24-well plate for suspension culture, and the plates were moved from the incubator to monitor the formation of cell clumps in a well via a microscope for 3 days (Figure 3).

Each well was captured in 16 images to observe all cells present. In Figure 3A, the control condition exhibited 5 to 6 small clumps 24 hours after inoculation; 48 hours later, two large clumps formed, accompanied by a significant decrease in the number of single cells. By 72 hours, single cells were rare, replaced with numerous cell clumps. Therefore, transitioning LMH cells from an adherent state to a suspension culture significantly induced cell aggregation. In contrast, in the DS-supplemented medium, no cell clumps were observed 24 hours after inoculation. At 48 hours, there were still no cell clumps, but several connected cells were observed (Figure 3B). By 72 hours, multiple cell clumps were identified, but a significant number of individual cells was present. The images obtained during the 3-day suspension culture in a DS-supplemented medium demonstrated that the addition of DS delayed cell aggregation of LMH cells.

### Effect of dextran sulfate on the transcription levels of cell-aggregation-related genes

To investigate the effect of DS in the media on the expression levels of genes involved in cell-cell interaction and cell survival, mRNA levels of such genes were quantitatively analyzed using the cells 72 hours post-inoculation, as in Figure 3.

**Figure 4:**
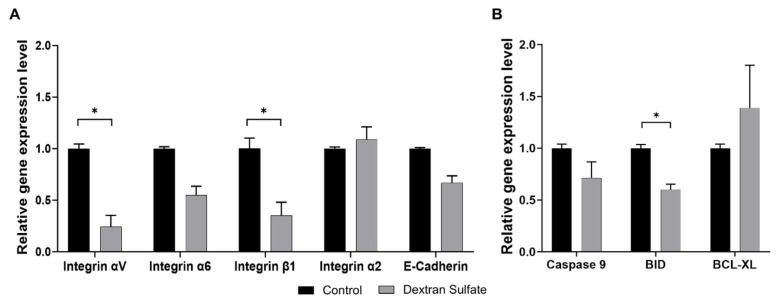
Relative expression levels of cell adhesion-related and apoptosis/survival-related genes of LMH cells in suspension cultures. Differential mRNA levels of the LMH cells on day 3 in 24-well plate suspension cultures were analyzed by quantitative real-time polymerase chain reaction; control medium (black-colored bar), DS-supplemented medium (grey-colored bar). (A) Genes related to cell adhesion: integrin αV, integrin α6, integrin β1, integrin α2, and E-Cadherin. (B) Genes related to pro-apoptosis (Caspase 9, BID) and anti-apoptosis (BCL-xL). Error bars indicate standard deviation calculated from three independent experiments. LMH, leghorn male hepatoma; DS, dextran sulfate; BID, BH3 interacting-domain death agonist. Multiple-paired t-tests was performed to analyze statistical significance (* p<0.05).

The expression levels of integrin family proteins (which play a crucial role in integrin-dependent cell adhesion) were analyzed (Figure 4A). Integrins are heterodimeric cell-binding transmembrane proteins composed of alpha and beta subunits. Interestingly, adding DS in the media decreased the expression of integrin αV, α6, and β1, while slightly increasing the integrin α2 expression level. The reduction in integrin expression can account for diminished cell aggregation since integrins facilitate the interactions between cells and the extracellular matrix (ECM) [37]. Consequently, it is expected that DS treatment on LMH cells will induce associations of various integrin subunits and trigger divergent signaling pathways from regular cellular differentiation. Additionally, expression of E-cadherin, a marker of epithelial cells, decreases with DS treatment, as expected. Overall, these results confirmed that DS supplementation decreased the characteristics of epithelial cells, possibly due to cell aggregation.

To identify whether the enhanced cell survival observed with DS addition in suspended LMH cells is associated with the regulation of genes involved in cell survival and apoptosis, the expression levels of Caspase 9, BID, and Bcl-xL were analyzed (Figure 4B). As expected, DS addition decreased the expression of pro-apoptotic genes Caspase 9 and BID and increased the expression of the anti-apoptotic gene Bcl-xL, increasing cellular viability.

### Effect of dextran sulfate on long-term culture of leghorn male hepatoma cells

**Figure 5:**
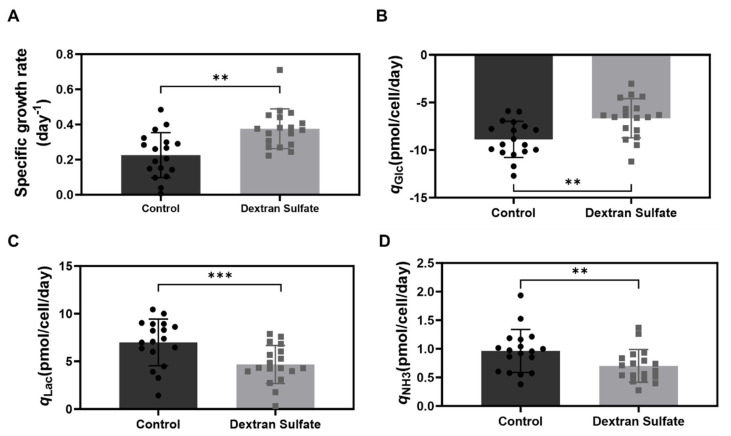
Comparative culture performance of LMH cells during 17 subculture passages between the control and DS-supplemented media. Adherently grown LMH cells were inoculated into suspension culture flasks containing 2 different media. Over a period of 2 months, cells were subcultured every 3 to 4 days, totaling 17 passages in 125 mL Erlenmeyer flasks. During each passage, (A) SGR; (B) specific glucose consumption rate (*q*_Glc_); (C) specific lactate production rate (*q*_Lac_); and (D) specific ammonia production rate (*q*_NH3_) were computed. An overall assessment of culture performance between the control and DS-supplemented media was conducted. The DS-supplemented medium exhibited higher SGR, decreased glucose consumption, and reduced lactate and ammonia production compared to the control medium. LMH, leghorn male hepatoma; DS, dextran sulfate; SGR, specific growth rate. Paired t-test was performed to analyze statistical significance (** p<0.01, *** p<0.005).

To investigate the impact of DS in the medium on the long-term culture performance of LMH cells (i.e., cell growth, viability, and metabolic processes), consecutive subcultures of LMH cells were performed every 3 to 4 days for 2 months using an initial inoculation of 0.3×10^6^ cells/mL in the control and DS-supplemented media conditions. Despite the suspension culture of LMH cells for 2 months, there was no reduction in cell aggregation in either of the media, suggesting that neither was suitable for adaptation of LMH cells to suspension culture (data not shown). Consequently, there was a challenge in precisely measuring and controlling cell inoculation densities due to the presence of aggregated cell clumps. Despite fluctuations in cell density, the statistical processing of cell culture data over 18 passages consistently demonstrated that the addition of DS improved the cell growth profile (Figure 5), which is consistent with the results shown in Figure 2. The average VCD and viability at day 3 or day 4 in the DS-supplemented medium increased by 16% and 13%, respectively, resulting in an improvement of 66% in the average cell growth rate (Figure 5A). Compared to the control condition, the DS-supplemented medium decreased glucose consumption rate by 24%, along with decreases of 33% and 27% in lactate and ammonia production rates, respectively (Figure 5B–5D). These findings are consistent with those depicted in Figure 2 and demonstrate the beneficial effects of DS on LMH cell growth and simultaneous reduction of cell aggregation during long-term suspension culture. However, the persistence of cell aggregation even after 2 months of cultivation indicates that the DS in the growth medium is not sufficient for adaptation of LMH cells to suspension culture.

## DISCUSSION

We evaluated the impact of DS supplementation in the suspension culture media on culture performance and cell aggregation of LMH cells used for vaccine production. LMH cells initially grown in attached cultures were inoculated into a suspension flask with a medium supplemented with DS. Adding DS decreased the size and number of cell clumps and enhanced culture performance. In addition, reduced cell-cell adhesion by DS addition was coordinated with biological responses, such as changes in gene expression. While cell aggregation decreased in the DS-supplemented medium, the formation of cell clumps could not be completely resolved even after 2 months of continuous subculturing.

DS, an anionic polysaccharide sulfate, is known to mitigate cell aggregation in various animal cell models by disrupting the interactions between cells and ECM [29–34]. The ECM is a structural and functional network that surrounds cells and consists of proteins, polysaccharides and proteoglycans. Importantly, ECM provides binding sites for cell adhesion molecules (CAMs) such as integrins, cadherins, selectins, and membrane proteins, which play crucial roles in the process of cell aggregation [38]. The negative charge of DS can create electrostatic repulsion with the negative charges of polysaccharides in the ECM, such as heparan sulfate proteoglycan, interfering with the interactions between the ECM and binding proteins [26,28]. The physicochemical changes in this ECM seem to affect signaling pathways that modulate the expression of adhesion proteins, influencing cell growth and survival. In this study, the addition of DS resulted in decreased expression of adhesion proteins, particularly E-cadherin and integrins (αV, α6, and β1), alongside enhancements in cell growth and viability. It is still unclear whether physicochemical stimuli directly induce signal transduction for promoting cell growth or whether a reduction in cell clump size improves cell growth by enhancing nutrient and oxygen supply to the cells inside the clumps. More investigation is required to comprehend the signaling mechanisms occurring in the ECM.

The mechanism of aggregation reduction by adding DS to the medium has similarities and differences with that of epithelial-mesenchymal transition (EMT), where epithelial cells, characterized by strong cell-cell adhesion, undergo a transition to mesenchymal cells, which exhibit enhanced mobility due to loss of cell-cell adhesion [39]. While DS supplementation and EMT differ in their induction mechanisms—DS disrupts interactions between molecules in the ECM, while EMT is driven by transcription factor-mediated gene expression regulation—both processes involve altering the expression of CAMs and remodeling the composition of the ECM to regulate cell aggregation [32,39]. As mentioned above, DS addition led to a decrease in epithelial markers such as E-cadherin and integrin αV, α6, and β1, which are typical CAMs expression patterns during EMT. However, integrin α2, which is important for collagen interaction and is reported to decrease in EMT, was slightly increased by DS addition. Therefore, both EMT and DS addition seem to influence CAM expression and cell aggregation, but they may induce different physiological responses in a context-dependent manner.

In this study, long-term subcultures were performed in DS-supplemented medium to adapt LMH cells to suspension culture (Figure 5). Suspension adaptation means transitioning adherent-derived cells to grow as individual cells without clumping in suspension flasks. Unfortunately, LMH cells continued to form cell clumps even after 2 months of subculturing in DS-supplemented media, while the level of clump formation was lower compared to the control condition. This result indicates that DS supplementation alone is insufficient for achieving successful adaptation of LMH cells to suspension culture. The suspension adaptation of mammalian cells can be influenced by various factors, including the inherent characteristics of the cells, the composition of the suspension medium, and the adaptation methods to suspension culture [24,40]. Among them, this study may have limitations in terms of suspension media composition. For example, the FBS utilized in the suspension culture medium in this study may have a negative effect on the suspension adaptation. While FBS contains numerous components essential for cell growth, such as growth factors and vitamins, it may also contain components like fibronectin and laminin, which can promote cell aggregation [41]. Hence, suspension culture in serum-free media might have better adaptation performance, as observed in a few previous studies on adaptation to suspension culture for certain animal cells or adopting a sequential approach involving serum reduction before transitioning to suspension culture [42,43]. In summary, while the addition of DS to the medium demonstrated a clear effect in reducing cell aggregation during the transition of LMH cells to suspension culture, it may be necessary to consider additional factors for complete adaptation to suspension culture.

In conclusion, we explored the impacts of DS supplementation on the culture performance, aggregation behavior, and long-term adaptation of LMH cells in suspension culture. While many animal cell lines are being utilized for vaccine production hosts, establishing an LMH cell line as an effective host capable of efficient large-scale production would expedite the supply of vaccines and treatments for various diseases that are threatening both to poultry and to many other animals. Furthermore, it could offer significant potential for overcoming human health crises.

## Figures and Tables

**Table 1:** Primer sequences used in quantitative real-time reverse-transcription polymerase chain reaction analysis

Gene	Primer sequence (5′-3′)		Role
*GAPDH*	F R	CCACATGGCATCCAAGGAGT GAACTGAGCGGTGGTGAAGA	Reference gene
*β-Actin*	F R	ATCTTTCTTGGGTATGGAGTC GCCAGGGTACATTGTGG	Reference gene
*Integrin αV*	F R	CCTACACCATGGGAGGGGTA GCAGCCACAGTCCAAGATCT	Cell adhesion
*Integrin α6*	F R	GCTGGAAACATGGACCTGGATAA TTCAGGTCAAGTTTGTCAGGCTGTA	Cell adhesion
*Integrin β1*	F R	TGTTTGTGGGGACCAGATTG CCAGGTGACATTTCCCATCA	Cell adhesion
*Integrin α2*	F R	CGCAGACAACAGGAGTCCTC AGCATTATTTGTGGCCGTGC	Cell adhesion
*E-cadherin*	F R	GACAGGGACATGAGGCAGAA GCCGTGACAATGCCATTCTC	Cell adhesion
*CP9*	F R	CCGAAGGAGCAAGCACG AGGTTGGACTGGGATGGAC	Pro-apoptotic
*BID*	F R	CTGTGAAAGGGAAGGCAGAG GCTACCAAAAAGGAGAGGGAA	Pro-apoptotic
*Bcl-xL*	F R	CTTTCAGCGACCTCACCTC ACAATGCGTCCCACCAGT	Anti-apoptotic

**Table 2 t2-ab-24-0372:** Values of C_t_ and delta C_t_ in quantitative real-time reverse-transcription polymerase chain reaction data analysis

Gene			C_t_ value	ΔC_t_ value	p-value
*Integrin αV*	Integrin αV	Control	24.061	3.218	0.03*
		Dextran sulfate	28.875	5.326	
*Integrin α6*	Integrin α6	Control	23.789	2.945	0.1
		Dextran sulfate	27.371	3.822	
*Integrin β1*	Integrin β1	Control	23.935	3.478	0.01*
		Dextran sulfate	28.333	5.032	
*Integrin α2*	Integrin α2	Control	28.040	7.196	0.52
		Dextran sulfate	30.626	7.077	
*E-Cadherin*	E-Cadherin	Control	23.121	2.277	0.1
		Dextran sulfate	26.409	2.860	
*Caspase 9*	Caspase 9	Control	24.751	4.294	0.28
		Dextran sulfate	28.096	4.795	
*BID*	BH3 interacting-domain death agonist	Control	24.363	3.519	0.01*
		Dextran sulfate	27.804	4.255	
*BCL-xL*	BCL-xL	Control	26.470	5.626	0.43
		Dextran sulfate	28.733	5.184	
